# Acid shock of *Listeria monocytogenes* at low environmental temperatures induces *prfA*, epithelial cell invasion, and lethality towards *Caenorhabditis elegans*

**DOI:** 10.1186/1471-2164-14-285

**Published:** 2013-04-27

**Authors:** Klaus Neuhaus, Peter Satorhelyi, Kristina Schauer, Siegfried Scherer, Thilo M Fuchs

**Affiliations:** 1 Lehrstuhl für Mikrobielle Ökologie, Department für biowissenschaftliche Grundlagen, Wissenschaftszentrum Weihenstephan, and ZIEL, Abteilung Mikrobiologie, Technische Universität München, Weihenstephaner Berg 3, Freising, 85350, Germany; 2 Present Address: IVAX Drug Research Institute, Berlini u. 49, Budapest, 1045, Hungary; 3 Present Address: Lehrstuhl für Hygiene und Technologie der Milch, Tiermedizinische Fakultät der Ludwig-Maximilians-Universität München, Schönleutnerstrasse 8, Oberschleißheim, D-85764, Germany

**Keywords:** *Listeria monocytogenes*, Virulence genes, Acidic pH, *prfA*, Invertebrates, Transcriptome, Global response, Invasion

## Abstract

**Background:**

The saprophytic pathogen *Listeria monocytogenes* has to cope with a variety of acidic habitats during its life cycle. The impact of low-temperature coupled with pH decrease for global gene expression and subsequent virulence properties, however, has not been elucidated.

**Results:**

qRT-PCR revealed for the first time a transient, acid triggered *prfA* induction of approximately 4-fold, 5.7-fold, 7-fold and 9.3-fold 60 to 90 min after acid shock of *L. monocytogenes* at 37°C, 25°C, 18°C, and 10°C, respectively. Comparable data were obtained for seven different *L. monocytogenes* strains, demonstrating that *prfA* induction under these conditions is a general response of *L. monocytogenes*. Transcriptome analysis revealed that the *in vivo*-relevant genes *bsh*, *clpP, glpD, hfq, inlA, inlB*, *inlE*, *lisR,* and *lplA1* as well as many other genes with a putative role during infection are transiently induced upon acid shock conducted at 25°C and 37°C. Twenty-five genes repressed upon acid shock are known to be down regulated during intracellular growth or by virulence regulators. These data were confirmed by qRT-PCR of twelve differentially regulated genes and by the identification of acid shock-induced genes influenced by σ^B^. To test if up regulation of virulence genes at temperatures below 37°C correlates with pathogenicity, the capacity of *L. monocytogenes* to invade epithelial cells after acid shock at 25°C was measured. A 12-fold increased number of intracellular bacteria was observed (acid shock, t = 60 min) that was reduced after adaptation to the level of the unshocked control. This increased invasiveness was shown to be in line with the induction of *inlAB*. Using a nematode infection assay, we demonstrated that *Caenorhabditis elegans* fed with acid-shocked *L. monocytogenes* exhibits a shorter time to death of 50% (TD_50_) of the worms (6.4 days) compared to infection with unshocked bacteria (TD_50_ = 10.2 days).

**Conclusions:**

*PrfA* and other listerial virulence genes are induced by an inorganic acid in a temperature-dependent manner. The data presented here suggest that low pH serves as a trigger for listerial pathogenicity at environmental temperatures.

## Background

The foodborne pathogen *L. monocytogenes* possesses a broad range of growth temperature (4°C to 45°C) and has been isolated from a variety of habitats including soil, decaying plants, water and animals [1]. This facultatively intracellular Gram-positive bacterium can cause systemic infections especially in immuno-compromised people with symptoms such as septicaemia, encephalomeningitis, placentitis and stillbirth. A main challenge for *L. monocytogenes* is the switch from a saprophytic lifestyle to a successful infection of mammals. Its capability to survive in low pH habitats such as fermented food including silage as well as in acidic host compartments like the stomach, the small intestine and phagosomes is an adaptation strategy common to both stages [2]. This aciduric capacity in turn raises persistent safety problems for the food industry [3]. On the other hand, stresses like acid are known clues for virulence gene regulation in many pathogenic organisms that may use the decrease in pH during stomach transit as signal to up regulate virulence genes [4,5]. However, the response of listerial virulence genes to acidic conditions under environmental temperatures, and a possible impact for the life cycle of this saprophyte, remains to be elucidated.

The response of *L. monocytogenes* to low pH involves the alternative sigma factor SigB (σ^B^) and is characterized by the synthesis of a number of proteins to mount a significant acid tolerance response (ATR). The ATR is capable of protecting cells against otherwise lethal acid stress conditions [6]. Several lines of evidence indicated that an adaptive acid tolerance response is required for pathogenicity of *L. monocytogenes*, and that the ability of this pathogen to survive gastric acid fluid and to invade host cells is directly linked to the activation of the ATR [7-9]. This is supported by the finding that the glutamate decarboxylase (GAD) system as the most important ATR component is required for listerial survival in the gastric environment [10], and that the deletion of LisRK, a two-component system (TCS) for signal transduction involved in the regulation of acid resistance, resulted in growth phase-dependent sensitivity to acid stress and a significant reduction in virulence [11]. A further protein linking a low pH-response to listerial virulence is the cysteine transport associated protein CtaP, which is necessary for growth in acidified BHI (pH 5.5) and for virulence after intragastric infection of mice [12].

The translation of PrfA, the master virulence gene regulator of *L. monocytogenes*, is regulated by post transcriptional mRNA-folding and blocked at temperatures below 30°C [13,14]. In accordance, it has been reported that *prfA* is not induced by low pH [15,16]. Interestingly, *L. monocytogenes* grown at 4°C or 37°C exhibited similar infection phenotypes upon intragastrical inoculation [17]. Furthermore, the production of PrfA and PrfA-dependent gene products was observed at low-temperature and within cells from the fruit fly *Drosophila melanogaster*[18,19]. This is in line with the demonstration that *L. monocytogenes* is pathogenic towards invertebrates such as *D. melanogaster*, the greater wax moth *Galleria mellonella* and the nematode *Caenorhabditis elegans* as model organisms for listerial pathogenicity [20-23]. These findings prompted us to investigate the putative connectivity between acid stress, low temperature gene induction, and virulence properties of *L. monocytogenes*.

Here we demonstrate by transcriptional analysis after treatment of *L. monocytogenes* with the inorganic acid HCl that *prfA* and other virulence factors are transiently induced by acidification at lower (10°C, 18°C, and 25°C), as well as at mammalian body temperature (37°C). We further show that σ^B^-influenced genes participate in the ATR also at 25°C, and that low pH increases epithelial cell entry of *L. monocytogenes* as well as its virulence towards *C. elegans*.

## Results

### Temperature-dependent induction of *prfA* after acid shock

**Figure 1:**
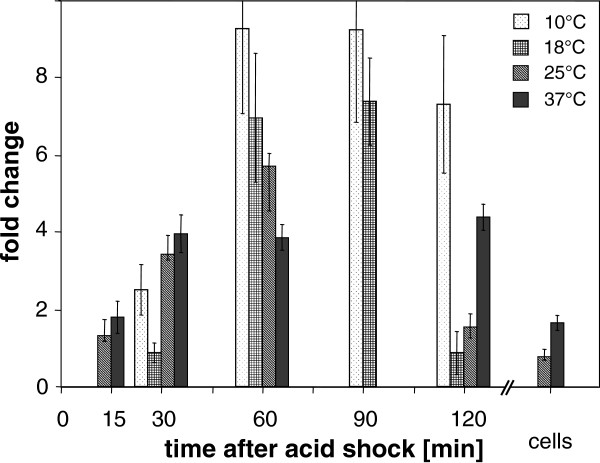
**Transcriptional induction of *prfA *upon acid shock at different time points.** Samples were taken before (t = 0) and at five time points until two hours after acid shock. Mean values of fold expression ± standard deviation calculated as ratio between *prfA* transcription after and before HCl treatment are presented. To obtain a sample of adapted cells, acid-shocked *L. monocytogenes* (120 min pH 5) were diluted and grown at pH 5.2 to OD_600nm_ = 0.5. Expression of *prfA* at 10°C and 18°C was measured by real time qRT-PCR in replicates of three and at 25°C and 37°C in two biologically independent experiments, respectively. The *prfA* induction was not determined at some time points (e.g., for 10 and 18°C at 15 min, 25 and 37°C at 90 min, and 10 and 18°C for the adaptation sample).

*L. monocytogenes* cultures grown to middle exponential phase were subjected to acid shock at different temperatures, and *prfA* transcript levels were examined with qRT-PCR. At 37°C, *prfA* showed approximately 4-fold higher transcript levels between 30 to 120 min after exposure to pH 5.0 with respect to the control. At 25°C, 18°C and 10°C, *prfA* was up regulated approximately 5.7-fold, 7.0-fold and 9.3-fold at 60 min after acid shock, respectively. *prfA* mRNA decreased after acid adaptation at 25°C and 37°C to nearly the same transcriptional level obtained before acid shock (Figure 1). Remarkably, the lower the tested temperature, the higher was the fold induction in comparison with untreated cells at the same temperature. The relative amount of *prfA* transcript at 25°C with respect to 37°C was 2.6 before acid shock (data not shown). Thus, the induction of *prfA* at 25°C and 60 min after acid shock compared to 37°C without acid shock was 2.2. Similarly, for 18°C and time point 60 min, a 3.7-fold increase compared to 37°C under pre-shock conditions was observed.

**Figure 2:**
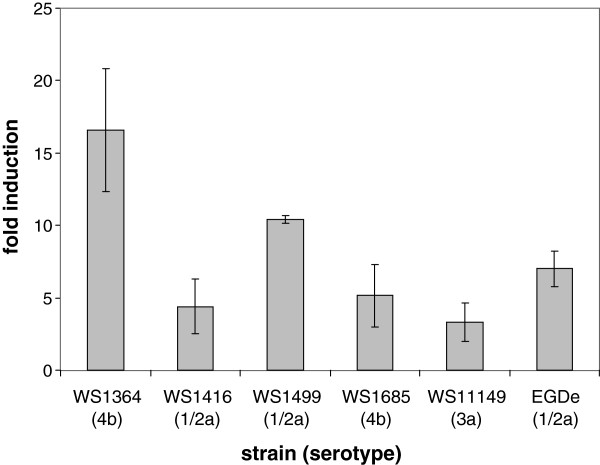
***prfA *induction levels in *L. monocytogenes *strains.** The transcriptional change of the *prfA* mRNA from five additional *L. monocytogenes* strains belonging to serotypes 1/2a, 3a and 4b at 18°C and 60 min after acid shock to pH 5.0 was measured by qRT-PCR in three independent experiments. The value for strain EGDe from Figure 1 is given in the last column as reference.

To exclude that this temperature-dependent *prfA* response to low pH is restricted to strain EGDe (serovar 1/2a), we examined six additional *L. monocytogenes* strains belonging to the serovars 1/2a, 3a and 4b for *prfA* transcription. For this purpose, the cultures were incubated for 1 h at pH 5.0 and 18°C, and their RNA was analyzed by quantitative real-time PCR (qRT-PCR). For five strains we received *prfA* induction levels of between threefold (WS11149) and 17-fold (WS1364) compared to unshocked bacteria (Figure 2). A correlation between serovar and induction levels could not be found. In one serovar 3a strain, WSLC 1211, no *prfA* transcription above basal level could be measured at neutral pH. However, the threshold cycle (CT) of RNA amplification with RNA from 18°C samples was the lowest in comparison with the other strains, suggesting a strong induction of *prfA* in WSLC 1211 after acid shock at low temperature. The 7-fold induction of *prfA* in strain EGDe corresponds to the mean fold induction value of 8.0 measured for the five additional *L. monocytogenes* strains. Taken together, these data demonstrate that the strong transcriptional increase found for *prfA* in strain EGDe under the described pH and temperature conditions is not a strain-specific phenomenon, but is generally observed in *L. monocytogenes*.

### Global transcriptional response upon acidification at 25°C and 37°C

**Figure 3:**
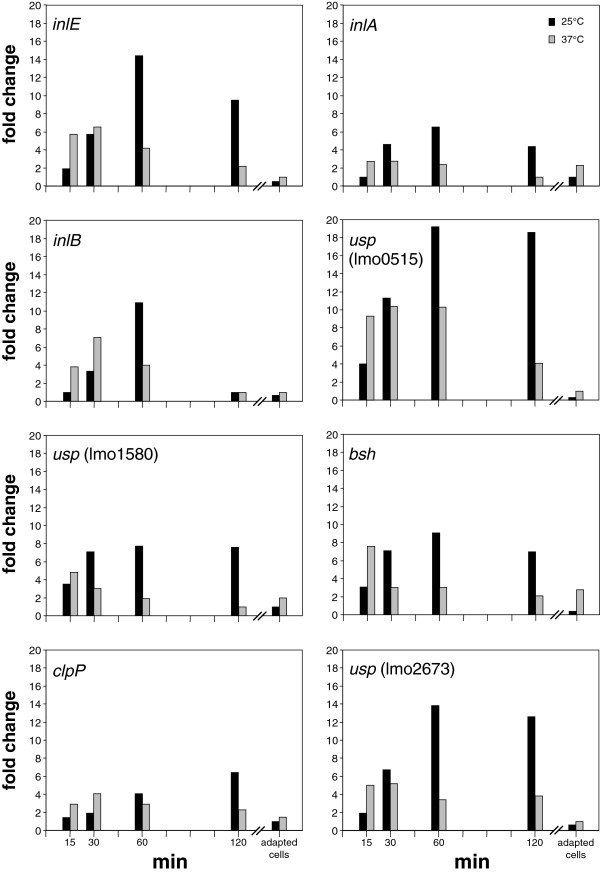
**Up regulation of internalin and other *in vivo*-relevant genes upon acid shock.** Transcriptional induction of selected genes transcriptionally induced after acid shock at 25°C (black columns) compared to acid shock at 37°C (grey columns) as deduced by microarray analysis. All genes have experimentally been demonstrated to play a role in listerial virulence (Additional file 2). Samples were taken 15, 30, 60 and 120 min after acid shock, and from acid-adapted cells. Two biological and two technical replicates were performed on microarray for each time point measured.

To assess the global impact of acid shock in gene induction, transcription profiling of *L. monocytogenes* was performed at 15, 30, 60, and 120 min post acid shock using microarray analysis. At 60 min after acid shock, 338 genes were found to be significantly up regulated at 25°C, and 383 genes at 37°C (Additional file 1). To attribute these experimental results to virulence properties, we conducted a comprehensive literature search for *L. monocytogenes* genes that are required for full virulence *in vivo*. We considered a gene to play a role during infection if its mutation showed attenuation in a mouse model (Additional file 2). Of these genes, we found 15 to be up regulated after acid shock at both 25°C and 37°C as summarized in Table 1. The genes mainly belong to the categories metabolism, regulation, stress and adhesins, and encode the following factors: bile salt hydrolase (Bsh), an ABC transporter (lmo0137), lipoate protein ligase A (*lplA1*), the proteolytic subunit of Clp protease (*clpP*), the RNA-binding protein Hfq, the TCS response regulator LisR, a PerR-like regulator (lmo1683), the response regulator DegU, three homologs of universal stress proteins (Usps; lmo0515, lmo1580, lmo2673), a hypothetical protein (SepA) and three internalins (InlA, InlB, InlE). Some of these genes, namely *inlA*, *inlB*, *inlE*, *bsh*, *clpP* and the three *usp* genes showed a significantly higher transcription rate at 25°C in comparison to 37°C at most time points after acid shock (Figure 3). Interestingly, their transcription ratios reverted to pre-shock levels after adaptation to low pH. Additionally, we noted induction of *frvA*, *btlB* and *purB* to be up regulated upon acid shock at 25°C, but not at 37°C (Table 1). These genes encode a Fur-regulated virulence factor, a factor similar to bile acid dehydratase, and an adenylosuccinate lyase. We also found *zinA* and *fbpA* to be up regulated at 37°C, but not at lower temperature.

**Table 1:** **List of genes required for full virulence*in vivo*that are induced after acid shock at 25 and 37°C**

**Locus**	**Gene**	**Functional annotation or prediction**	**Comments**^**a**^	**T**	**Min after acid shock**				**Adaptation**^**b**^
					**15**	**30**	**60**	**120**	
**Induced after acid shock at both 25°C and 37°C.**									
lmo0137		ABC transporter	Required for full virulence in mice [24]	25°C	4.0	5.0	5.8	9.5	0.8
				37°C	19.2	5.7	7.9	6.3	n.a.
lmo0264	*inlE*	InlE, internalin E	Required for full virulence in mice [25]	25°C	1.9	5.7	14.4	9.5	0.5
				37°C	5.7	6.5	4.2	2.2	1*
lmo0433	*inlA*	InlA, internalin A	Required for host cell entry *in vivo*[26], PrfA-regulated [27]and σ^B^[28]	25°C	1*	4.6	6.5	4.4	1*
				37°C	2.7	2.8	2.4	1*	2.3
lmo0434	*inlB*	InlB, internalin B	Required for host cell entry *in vivo*[26], σ^B^-regulated [28]	25°C	1*	3.3	10.9	1*	0.7
				37°C	3.8	7.1	4.0	1*	1*
lmo0515		Universal stress protein	Important for growth *in vivo*[29]	25°C	4.0	11.3	19.2	18.6	0.3
				37°C	9.3	10.4	10.3	4.1	1*
lmo0931	*lplA1*	Lipoate protein ligase A	Aborted replication in macrophages and *in vivo*[30]	25°C	1*	1*	1.7	2.3	1.5
				37°C	1.9	2.1	2.4	2.5	1*
lmo1295	*hfq*	RNA-binding protein	Contributes to virulence [31]	25°C	1*	2.1	3.4	2.8	0.6
				37°C	1*	3.7	2.8	1.7	1*
lmo1377	*lisR*	TCS response regulator	Contributes to virulence [11,32]	25°C	1*	0.9	1.7	2.9	1*
				37°C	1*	1*	2.0	2.0	1.3
lmo1580		Universal stress protein	Important for growth *in vivo*[29]	25°C	3.5	7.1	7.7	7.6	1*
				37°C	4.8	3.0	1.9	1*	2.0
lmo1683	*perR*	Transcription regulator (Fur family), PerR in *B. subtilis*	Mutant is attenuated in mice [33]	25°C	1*	2.6	3.5	5.4	1*
				37°C	1.6	4.0	5.1	4.6	2.3
lmo2067	*bsh*^**c**^	Bile salt hydrolase	PrfA- [34] and σ^B^-regulated [35], necessary for gastrointestinal persistence [36] and virulence [37]	25°C	3.1	7.1	9.1	7.0	0.4
				37°C	(7.6)	(3.0)	(3.0)	(2.1)	(2.8)
lmo2157	*sepA*	Hypothetical protein	Intracellularly up regulated [38,39], deletion decreases virulence [40]	25°C	1*	2.8	5.9	5.4	1*
				37°C	3.2	5.0	4.3	1.6	1*
lmo2468	*clpP*	ATP-dependent Clp protease, proteolytic subunit	Required for full virulence [41] and induced intracellularly [38]	25°C	1.4	1.9	4.1	6.4	1*
				37°C	2.9	4.1	2.9	2.3	1.5
lmo2515	*degU*	Response regulator	Contributes to virulence [42]	25°C	0.9	0.9	2.0	3.0	1*
				37°C	1*	2.3	2.9	1.9	1*
lmo2673		Universal stress protein	Important for growth *in vivo*[29]	25°C	1.9	6.7	13.8	12.6	0.6
				37°C	5.0	5.2	3.4	3.8	1*
**Induced after acid shock at 25°C, but not at 37°C**									
lmo0641	*frvA*	Fur-regulated virulence factor	A putative P-type ATPase required for virulence [43]	25°C	2.5	5.9	6.2	4.9	2.4
lmo0754	*btlB*	Bile acid 7-α-dehydratase	Important for intestinal persistence [36]	25°C	1.9	2.0	1.9	1.5	1*
lmo1773	*purB*	Adenylosuccinate lyase	Mutants attenuated for systemic infection in mice [44]	25°C	1.0	0.9	2.4	1*	1*
**Induced after acid shock at 37°C, but not at 25°C**									
lmo0153	*zinA*	Zinc ABC transporter, Zn-binding	Contributes to full virulence *in vivo*[45]	37°C	1*	1*	1.85	2.28	1.38
lmo1829	*fbpA*	Homologies to atypical fibronectin-binding proteins	Increases adherence, required for intestinal and liver colonization [46]	37°C	1*	2.0	1.8	2.3	1*

Fold induction values: The integer digits (up regulated genes) or the first post decimal digit (down regulated genes) is significant (p < 0.05).

n.a.: data not available; T, temperature.

1*: No significant difference (p > 0.05) between experiment and control; the value was therefore set to 1.

^**a**^Comments are based on the given reference(s).

^**b**^The pH for adaptation was 5.2 instead of 5.0 as for all other time points, to allow for significant growth at low pH.

^**c**^The 37°C values for *bsh* did not pass the quality threshold due to insufficient number of independent data points; the data available are in brackets.

We identified 33 additional genes by our approach that have not been demonstrated to play a role *in vivo*, but are required for epithelial cell invasion or intracellular replication, are induced during intracellular replication, or are influenced by σ^B^, VirR or PrfA (Table 2). Two of these genes code for internalin-homologs.

**Table 2:** **List of genes identified in this study that are influenced by PrfA,** σ^B^**, or VirR, are required for epithelial cell invasion or intracellular replication, are induced during intracellular replication, or are homologs of internalin**

**Locus**	**Gene**	**Functional annotation or prediction**	**Comments from literature**	**T**	**min after acid shock**				**Adaptation**^**1**^
					**15**	**30**	**60**	**120**	
**Induced after acid shock at both 25°C and 37°C**									
lmo0206		Hypothetical protein	PrfA-influenced, up regulated intracellularly [38]	25°C	1*	2.0	3.2	3.1	0.9
				37°C	1.4	4.1	2.4	1*	1*
lmo0223	*cysK*	CysK, highly similar to cysteine synthase	PrfA-box upstream, cell wall proteome [47]	25°C	2.3	6.2	6.6	4.1	n.a.
				37°C	2.5	3.9	2.5	2.9	2.0
lmo0593		Nitrate transporter	Up regulated intracellularly [38]	25°C	0.6	1*	1.9	2.2	0.4
				37°C	6.2	5.7	6.6	2.9	1*
lmo0596		Hypothetical protein	Contains PrfA- and RpoS-boxes [48], σ^B^-dependent [35]	25°C	0.8	1.7	2.0	1.7	0.5
				37°C	10.1	5.9	4.6	3.3	5.7
lmo0610	*inl*	Peptidoglycan bound protein (LPXTG motif)	Internalin-homolog [49]	25°C	1.5	3.7	6.4	5.7	0.4
				37°C	9.1	5.9	5.4	1*	1*
lmo0669		Oxidoreductase	σ^B^[50] and PrfA-regulated [48], required for intracellular survival [51]	25°C	1.8	4.4	8.3	7.7	0.7
				37°C	3.6	5.7	5.0	3.7	3.8
lmo0759		Lactoylglutathione lyase	Mutant is intracellularly attenuated [39]	25°C	2.6	4.9	8.2	11.0	0.8
				37°C	4.8	4.9	4.5	2.9	1*
lmo0783		Mannose-specific PTS component IIB	σ^B^-dependent [52]	25°C	0.7	1*	2.2	2.0	0.8
lmo0880		Cell wall associated protein precursor (LPXTG motif)	Up regulated intracellularly [38], σ^B^-regulated [53]	25°C	2.4	6.9	9.1	8.8	1*
				37°C	50.1	18.8	12.5	23.9	17.4
lmo1138	*clpP*	Proteolytic component of Clp protease	Up regulated intracellularly [38]	25°C	n.a.	1.8	3.4	4.5	n.a.
				37°C	2.0	4.5	2.8	1.4	n.a.
lmo1293	*glpD*	Glycerol-3-phosphate dehydrogenase	Up regulated intracellularly [38]	25°C	n.a.	4.9	7.7	5.9	n.a.
			Required for intracellular growth [39]	37°C	5.7	4.4	3.3	5.3	7.0
lmo1302	*lexA*	SOS response regulator	Up regulated intracellularly [38]	25°C	1.8	2.8	3.5	5.0	1*
				37°C	2.0	2.4	3.5	2.2	1*
lmo1416		Hypothetical protein	Deletion renders *L. monocytogenes* bile sensitive [36]	25°C	1*	1.7	7.8	11.5	4.7
				37°C	1*	8.9	7.7	9.4	5.9
lmo1734	*gltS*	Glutamate synthase (large subunit)	Intracellularly up regulated [39]	25°C	1.7	2.0	5.2	1*	1*
				37°C	1*	6.9	6.0	1*	2.4
lmo1991	*ilvA*	Threonine dehydratase	Involved in intravacuolar survival [38], intracellularly up regulated [39]	25°C	1.7	2.1	2.9	3.1	0.9
				37°C	2.7	3.3	3.8	2.9	1*
lmo2085		Peptidoglycan bound protein (LPXTG motif)	Up regulated intracellularly [38], σ^B^-dependent [53]	25°C	1.7	6.3	9.4	7.3	0.7
				37°C	6.1	5.8	3.9	4.2	1*
lmo2156		Hypothetical protein	VirR regulated [54]	25°C	8.3	18.0	11.1	4.6	2.0
				37°C	6.2	9.9	4.6	4.6	1.7
lmo2191	*spxA*	Hypothetical protein	Up regulated intracellularly [38]	25°C	1*	1.9	3.6	3.6	0.8
				37°C	6.2	4.7	4.1	4.1	2.8
lmo2199	*ohrA*	Hypothetical protein	Involved in hydroxyperoxide resistance [55], up regulated intracellularly [38]	25°C	1*	1.7	3.4	7.4	1.5
				37°C	1.5	2.1	3.8	3.9	1*
lmo2200	*ohrR*	Transcription regulator	Involved in hydroxyperoxide resistance [55], up regulated intracellularly [38]	25°C	1*	2.5	5.2	10.2	2.0
				37°C	9.8	11.2	11.2	14.0	3.4
lmo2206	*clpB*	similar to endopeptidase Clp ATP-binding chain B	chaperone, up regulated intracellularly [38,39]	25°C	2.9	5.8	5.8	4.1	n.a.
				37°C	7.2	6.9	3.5	1.5	n.a.
lmo2434	*gadD3 /gadD*	Glutamate decarboxylase	Up regulated during acid stress [15] and intracellularly [39], σ^B^-dependent [56]	25°C	1*	5.7	10.1	11.1	0.4
				37°C	1.8	2.5	2.4	1*	1.5
lmo2570		Integral membrane protein	PrfA-regulated [48]	25°C	0.7	2.5	5.8	7.6	0.8
				37°C	2.1	4.1	3.4	2.7	1*
lmo2571		Nicotinamidase	PrfA-regulated [48]	25 °C	0.5	4.1	3.4	2.7	1*
				37°C	12.6	3.6	8.2	10.1	1*
lmo2572		Dihydrofolate reductase, chain A	PrfA-regulated [48]	25°C	1*	3.9	8.6	9.1	1*
				37°C	5.5	3.9	n.a.	3.7	2.3
lmo2573		Zinc-binding dehydrogenase	PrfA-regulated [48]	25 °C	2.3	6.8	10.6	10.0	0.5
				37°C	14.3	6.1	6.2	4.3	6.5
lmo2695		Dihydroxyaceton kinase	Mutant attenuated in macrophages (Fuchs *et al*., unpublished data)	25°C	1*	5.5	8.2	5.9	0.8
				37°C	2.8	2.6	2.6	2.8	2.1
lmo2696		Dihydroxyaceton kinase	Mutant attenuated in macrophages (Fuchs *et al*., unpublished data)	25°C	n.a.	5.3	13.5	18.6	n.a.
				37°C	1.8	5.5	3.2	2.3	1.7
lmo2713		Secreted protein with GW repeat	Up regulated intracellularly [38], σ^B^-regulated [48]	25°C	1.5	3.0	5.0	3.9	1*
				37°C	3.1	2.5	4.0	2.8	1.5
**Induced after acid shock at 25°C, but not at 37°C**									
lmo1538	*glpK*_*1*_	Glycerol kinase	Up regulated intracellularly [39]	25°C	4.3	3.5	2.5	1.5	4.6
**Induced after acid shock at 37°C, but not at 25°C**									
lmo0232	*clpC*	Endopeptidase Clp, ATP-binding chain C	Plays a role in adhesion and invasion, and modulates the expression of InlA, InlB and ActA [57]	37°C	2.7	9.6	6.0	2.0	1.8
lmo1439	*sod*	Superoxide dismutase	Mutant attenuated in survival within macrophages [58]	37°C	1*	1*	2.5	2.7	2.0
lmo2250	*arpJ*	ABC-transporter for arginine	Mutant attenuated in intracellular growth [59]	37°C	1*	2.3	2.0	2.0	1*

See legend of Table 1.

Acid shock is characterized by a mainly posttranslational activation of σ^B^ in Gram positive bacteria [60]. Accordingly, no induction of *sigB* transcription was observed in the microarray experiments. However, as demonstrated by the induction of σ^B^-influenced genes (lmo0433, lmo0434, lmo0596, lmo0669, lmo0783, lmo0880, lmo0913, lmo1295, lmo2067, lmo2085, lmo2434, lmo2713; Tables 1 and 2), σ^B^ seems to be activated after acid shock. Furthermore, we observed down regulation of several genes belonging to the *rsb*-operon after acid shock, namely *rsbR*, *rsbS*, *rsbT*, and *rsbU*. The *rsbRSTU*-gene products are involved in the upstream switch module of the σ^B^ -regulation cascade [61]. Sixty min after acid shock at 25°C, a 5.9-fold, 3.6-fold, 5.3-fold, and 4.8-fold down regulation of these genes was recorded. Their expression levels were restored to pre-shock values when acid adaptation had taken place (data not shown).

**Figure 4:**
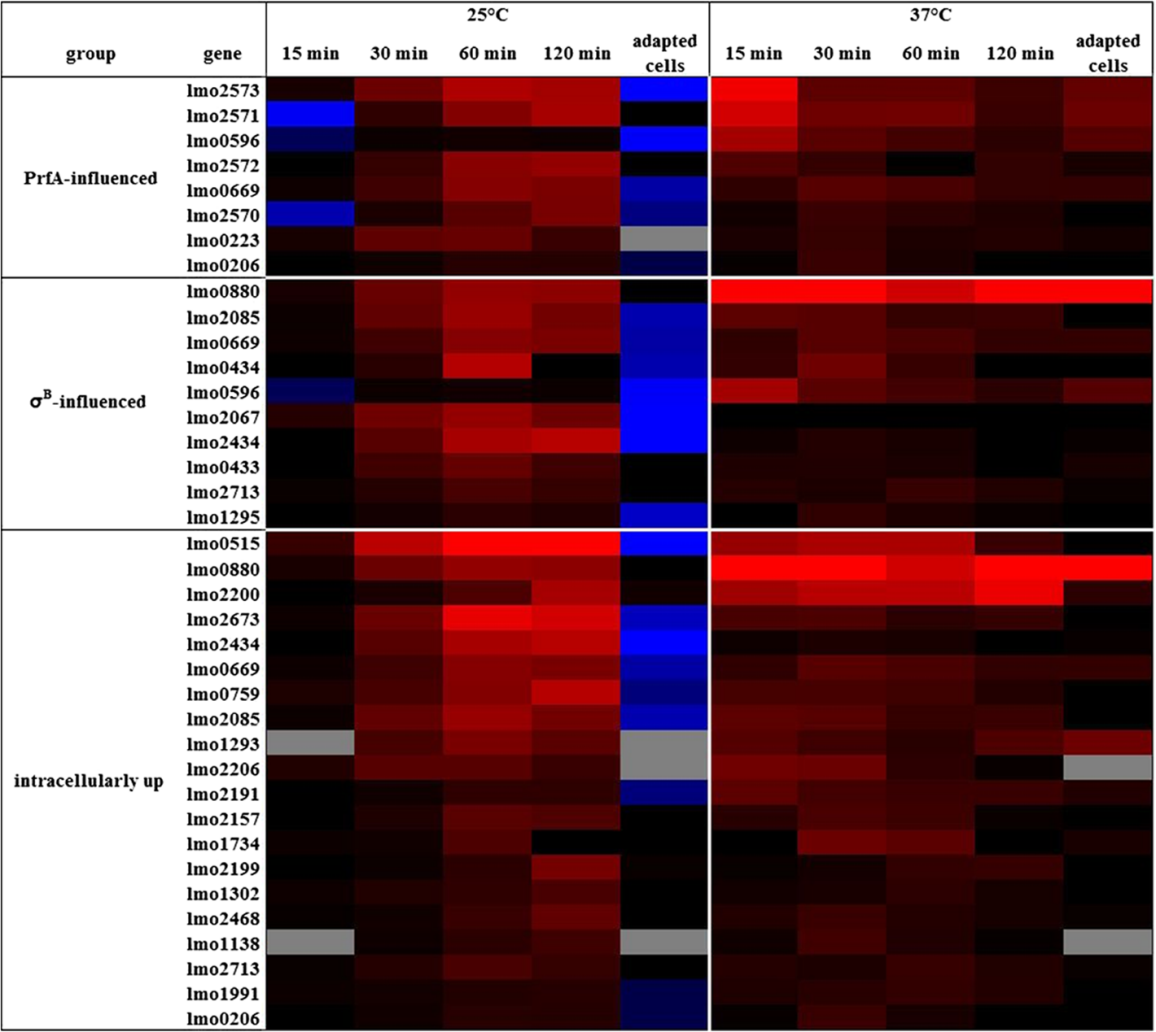
**Heat map of genes influenced by PrfA or σ**^**B**^**, or of intracellularly induced genes.** Average expression of *in vivo*-relevant expression after acid shock at 25°C and at 37°C. In both experimental settings, samples were taken 15, 30, 60 and 120 min after acid shock, and from acid-adapted cells. Genes have been grouped according to their putative regulation by PrfA and σ^B^, as well as to up regulation during intracellular growth. Up regulation is colored in shades of red, down regulation in shades of blue. “No change” is indicated by black and missing values by grey coloring.

To summarize these findings, we grouped those genes into the categories of PrfA- or σ^B^-influenced or intracellularly up regulated genes. Genes in all three groups are up regulated to various degrees not only at body temperature, but also at lower environmental temperature (Figure 4). In contrast to the experiments performed at 37°C, most of the genes analyzed at 25°C reach their maximal expression 60 min or later post acid shock.

### Data validation

**Figure 5:**
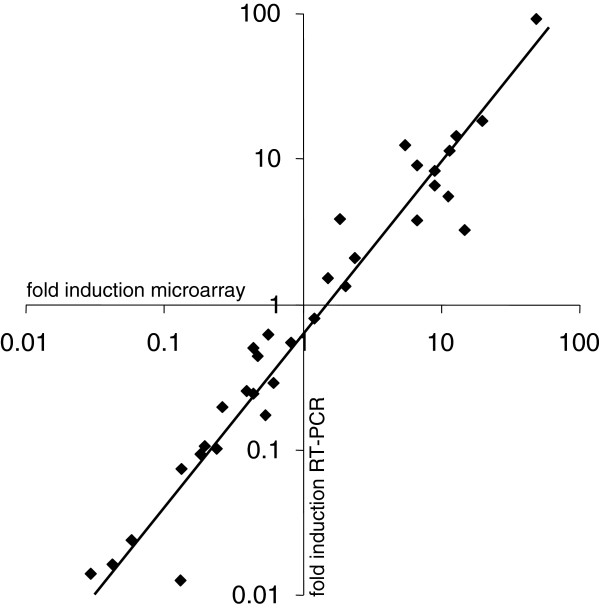
**Correlation of microarray data and qRT-PCR analysis of selected genes.** The following genes were selected for microarray validation by qRT-PCR: *prfA*, *hly*, *flhB, cheA*, lmo0109, lmo0847, lmo1740, *rsbR*, lmo1389, lmo1997, lmo2434, and lmo2784 (see Additional file 3 for more functional details). The validated genes checked at various time points (♦) displayed a significant correlation (level of correlation = 0.966) between the data obtained by microarray analysis and by qRT-PCR. Three independent qRT-PCR experiments were performed.

Although two biological and two technical replicates on each array were performed, twelve up and down regulated genes representing virulence and metabolic factors as well as regulatory genes and those involved in motility were chosen for qRT-PCR to further validate the microarray results (Additional file 3). This approach confirmed induced transcription of *prfA*, *hly*, *flhB,* and *cheA*, and a set of genes encoding the following predicted functions: a transcriptional regulator (lmo0109), glutamine ABC transporter (lmo0847 and lmo1740), a subunit of a sugar ABC transporter (lmo1389), the IIA component of a mannose-specific phosphotransferase system (PTS, lmo1997), a protein similar to glutamate decarboxylases (lmo2434), and a stress protein (lmo2784). Down regulation of *rsbR* (lmo0889) encoding a positive regulator of σ^B^-activity was also approved by qRT-PCR. 16S rRNA transcription was used for normalization. The values obtained correlated well with the respective microarray data (Figure 5). Calculation revealed a high level of concordance (r = 0.966), demonstrating a significant correlation between the induction or repression levels determined by microarrays and by qRT-PCR. The qRT-PCR analysis thus confirmed the microarray-derived expression data of the genes investigated.

### Listerial genes with lower transcript levels at 37°C and 25°C following acid shock

Twenty-five genes observed to be down regulated at both temperatures after acid shock have been mentioned in the literature to be intracellularly down regulated and/or to be negatively controlled by the virulence regulators PrfA, DegU, MogR or PerR (Additional file 4). Some of these genes code for proteins that are important for cell division and replication (*dnaA*, lmo0394, *minD*, *minC*, *divIVA*, *ftsZ*, *pbpB*, *ftsX*, *ftsE*, *murA*/*namA*, *cydA*). They all have in common to be down regulated during intracellular growth, a response that is also reflected by slower growth rates after acid shock (data not shown). Another group of genes (lmo0178, lmo0644, lmo0675, lmo1604, *plsX*, *dra*) are known to be repressed by PrfA, PerR, DegU, MogR, and Fri (Additional file 4), suggesting that these and other down regulated genes (listed in Additional file 4; *iap*, lmo0847, lmo0888, lmo0970, *pycA*, lmo1081, lmo1084, lmo1254, *fabD*, *pbpB*, *oppA*, lmo2202, *lgt*) are not necessary within acidic host compartments. Another set of *in vivo*-relevant genes was observed in our study to be down regulated upon acid shock at 25°, but not at 37°C. These genes include *purA*, *inlH*, lmo0540/lmo2229/lmo2754 encoding penicillin-binding proteins, *flaA*, lmo0848 encoding an ABC transporter, *dal*, *clpE*, *racE*, *lpd*, *bilEA*, *zurR*, *zurM*, *lapB*, *mprF*, *gtcA*, and *ami* (Additional files 1 and 2). Their negative transcriptional response to low-temperature may indicate a predominant virulence role towards mammals rather than invertebrates. In contrary, the metabolic genes *pgl*, *lap* and *gap*, as well as *brtA* encoding a bile sensor (Additional files 1 and 2) are down regulated only at 37°C and might therefore contribute to pathogenicity towards invertebrates.

### Increased invasiveness of listerial cells acid-shocked at 25°C

**Figure 6:**
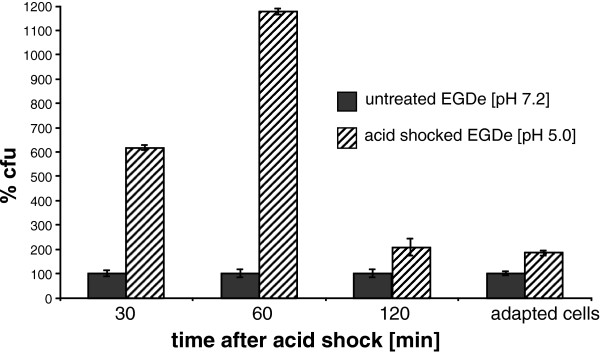
**Invasiveness of *L. monocytogenes *after acid shock at 25°C.** Invasion assays were performed with Caco-2 cells. Untreated and acid-shocked EGDe cells were used for infection at an MOI of 10. The invasion process was blocked after 1 h by cell washing and gentamycin treatment, and the epithelial cells were lysed to enumerate intracellular listeriae. The percentage of colony forming units (cfu) derived from acid treated inoculate as normalized to the control using untreated cells. Standard deviations of three independent invasion assays, performed at least as triplicates, are shown. In each experiment, the significance value was below 0.05 (p < 0.05) as calculated by the Student’s t-test. The fold difference in invasion is approximately 6-fold at 30 min and 12-fold at 60 min after acid shock.

To investigate the biological relevance of acid shock at low temperature on virulence gene induction in *L. monocytogenes*, bacteria were cultivated at 25°C in neutral BHI medium, well below the reported 30°C-threshold for *prfA* gene induction [13], and then transferred to BHI medium at pH 5.0 for acid shock as described. The efficiency of unshocked, acid-shocked and acid-adapted EGDe cells to enter Caco-2 cells was determined. For this purpose, the epithelial cell layer was incubated for one hour at a multiplicity of infection (MOI) of 10. Extracellular bacteria were then eliminated, and the number of intracellular cells was determined by plating host cell lysates. In comparison to bacteria grown at pH 7.2, a strong 6.2-fold and 11.7-fold increased invasiveness of listerial cells 30 and 60 min after acid shock was observed (Figure 6). *L. monocytogenes* cells adapted to low pH behaved similar to those grown in BHI medium at pH 7.2 as control. These data clearly indicate that the exposure to acidic pH at 25°C enhances transiently the ability of *L. monocytogenes* to invade colon epithelial cells.

### Acid-shocked *L. monocytogenes* reduce the lifespan of *C. elegans*

**Figure 7:**
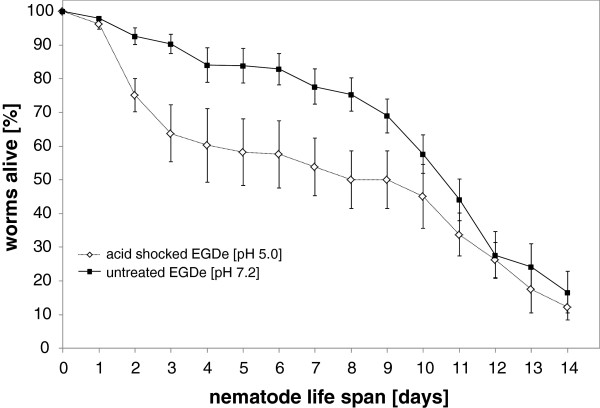
**Nematode infection assays.** Death curves of nematodes fed with acid-shocked *L. monocytogenes* (◊) and unshocked control cells (■). Surviving worms were transferred daily to freshly prepared acid-shocked or unshocked *L. monocytogenes* for up to 14 d. The error bars are from four independent experiments using 20 worms in each condition, totaling 160 worms.

The induction of *bsh* and the internalin genes *inlA*, *inlB* and *inlE* at acidic pH in combination with low temperature might point to a role of this listerial response in the environment. To further test this hypothesis, acid-shocked *L. monocytogenes* were fed to *C. elegans*. The nematode has already been demonstrated to be an established model to study pathogenicity mechanisms of *L. monocytogenes*[23]. *L. monocytogenes* were acid-shocked for 1 h at pH 5.0 and washed three times in fresh medium. Untreated EGDe cells served as control. Listerial cells were freshly prepared every day to serve as feed for the nematodes. The nematodes were kept at room temperature (22°C), and the number of viable and dead worms was monitored each day. The time point at which 50% of worms were dead (TD_50_) was calculated as 6.4 ± 0.5 days upon infection with acid-shocked *L. monocytogenes* and 10.2 ± 0.3 days when using unshocked cells (Figure 7). The significant reduction (p < 0.05) of the nematode’s life span by 3.8 days due to low pH shocked *L. monocytogenes* demonstrates the biological relevance of differential gene induction at environmental temperatures below 37°C as a trigger for listerial pathogenicity towards invertebrates.

## Discussion

Environmental conditions which *L. monocytogenes* is exposed to prior to host invasion are decisive for its infective potential as virulence gene expression is generally down regulated after cold shock or in rich medium [62,63]. In contrast, exposure to high temperature (42°C), oxidants, entry into stationary phase induces the transcription of virulence genes, or oxygen limitation increases invasiveness 100-fold [64]. Stimuli for *prfA* induction include body temperature, low iron concentration, high osmolarity, activated charcoal, oxidative stress and conditions simulating the gastrointestinal system [13,14,65-67], whereas fermentable sugars repress PrfA-dependent virulence genes [34,68].

Our finding of *prfA* induction upon acid shock contrasts the outcome of previous studies [15,68] whose experimental conditions, however, are not comparable to those used in our work. Cells had been taken from stationary phase or from cultures grown to exponential phase in the presence of low pH, thus allowing the cells to adapt to acid. Likewise, Garner *et al.*[69] reported an attenuated invasiveness of *L. monocytogenes* for Caco-2 cells, when the bacteria were grown at pH 5.5 in the presence of organic acids, and Rieu *et al*. [70] demonstrated a decrease in virulence gene transcription after 5 h at pH 4.0 achieved with acetic acid. In both studies, listerial cells had been adapted to low pH previous to the experiment. These results are in line with our data showing *prfA* transcription levels and listerial invasiveness of epithelial cells to decrease to nearly pre-shock levels after adaptation to low pH (Figures 1, 4 and 6). However, conducting a short time experiment, Werbrouck *et al.*[16] failed to detect *prfA* induction after 1 h acid shock to pH 5.5. This conflicting finding might be explained by their use of organic acids that are much more harmful to the bacteria. To mimic gastric conditions, we, in contrast, added inorganic HCl to acid shock *L. monocytogenes*. The observed changes do not reflect growth-phase dependent physiological changes because listerial growth after acid shock was not observed for at least 1 hour (data not shown); therefore, the transcriptional response appears acid-shock specific. The *prfA* inducibility by HCl at 37°C as demonstrated here might be considered as an imitation of conditions during the stomach passage where acid is an early signal for a possible host entry, thus preparing bacteria for infection [71].

We assume that in contrast to a temporary acid shock, prolonged mild acid conditions are often encountered by saprophytic bacteria in the environment, namely upon geological processes, fermentation of plant material, activity of plant roots or food processing [7,17,72]. As a result of this acid adaptation process, virulence gene expression probably decreases in *L. monocytogenes*. Due to the ATR, the acid adapted listeriae also show an enhanced capacity to survive the gastric barrier as reflected by higher survival rates 15 min after intragastric infection of mice [73]. In this case, a further signal for virulence gene induction occurs after stomach exit, namely exposure to short chain fatty acids accompanied with pH neutralization [74].

An unexpected result of this study was the induction of *prfA* and other *in vivo*-relevant genes at temperatures lower than 37°C after acid shock (Figures 1, 4). Johansson *et al.* reported that *prfA* mRNA is stable at 30°C, but PrfA is only formed at 37°C [13]. According to Loh *et al*. [75], PrfA expression is approximately 16-times lower at 30°C compared to 37°C and not detectable in *L. monocytogenes* cultivated at 20°C in BHI medium. However, using a *prfA*-promoter fusion to *gfp*, substantial expression of the reporter protein at 30°C in the heterologous system of *E. coli* was visible [75]. This suggests PrfA expression to be generally possible at temperatures below 37°C. Accordingly, it was argued that *prfA* upregulation occurs most likely at the σ^B^-dependent P2 promoter and not via the σ^A^-dependent P1 promoter, producing monocistronic *prfA* mRNA which is not blocked at temperatures below 30°C [34].

A remarkable high number of genes involved in virulence, controlled by one of the main virulence regulators or induced during intracellular growth, also positively responded to acid shock at both temperatures, indicating that this signal contributes to the adaptation of *L. monocytogenes* during infection. A differential temperature response was observed for few genes only (Tables 1 and 2), and we hypothesize that this finding points to their requirement in specific hosts. While *zinA*, *fbpA*, *clpC*, *sod* and *arpJ* at 37 °C might specifically be induced in mammalian hosts, the low temperature-induced genes *frvA*, *btlB*, *purB* and *glpK*_*1*_ may indicate a role in invertebrates. A set of up regulated genes identified here is influenced by σ^B^. σ^B^ is not only responsible for listerial survival under acid stress conditions [76], but for the transcription of several *in vivo*-relevant genes such as *bsh*, *inlAB* and *opuCA*[28,35,51,77]. Remarkably, σ^B^ contributes to virulence and gastrointestinal spread, but not systemic infection [78].

Uptake of *L. monocytogenes* into non-phagocytotic cells is mainly mediated by InlAB [27,28]. In agreement with the above reported increased invasiveness at 25°C, we observed a transient induction of *inlA* and *inlB* (e.g., at 60 min 6.5-fold for *inlA* and 10.9-fold for *inlB*; Figure 3), despite a normally lesser transcription at lower temperatures [19,79]. Enough InlAB seems to be produced for invasion after acid shock [57]. We detected a 12-fold increase in cell culture invasiveness one hour after acid shock at 25°C, compared to a modest 3.1-fold increase after acid shock at 37°C or at 30°C [9,80]. In contrast to acidic stress at 37°C [81], listerial cells adapted to acid lost their higher potential to invade Caco-2 cells. σ^B^, which is active at low environmental pH, contributes to invasion by controlling *inlAB*[28]. This is in accordance with our finding that σ^B^-dependent genes are induced upon acid shock.

The above described data prompted us to investigate acid shock induced *prfA* transcription at even lower temperatures of 18°C and 10°C. Unexpectedly, lower temperatures corresponded with higher folds of *prfA* induction when compared to unshocked cells at the same temperature (Figure 1). A similar response was observed for six other *L. monocytogenes* strains. We wondered whether the increase of virulence factor transcription due to acidic conditions at temperatures below 37°C confers increased pathogenicity of *L. monocytogenes* under yet unknown conditions. To answer this question, we applied a nematode killing assay. When we fed acid-shocked *L. monocytogenes* to *C. elegans* we could observe that the nematodes died faster compared to nematodes fed with unshocked bacteria. These observations clearly showed the biological relevance of virulence gene induction after acid shock at room temperature. *D. melanogaster* and its cells grown at 30°C have also successfully been introduced as a model for listerial infection [18,20]. Therefore, it could be hypothesized that other poikilothermal animals are hosts of *L. monocytogenes*, although virulence genes appear generally down regulated at low temperature [63].

Indeed, *Listeria* spp. have been isolated from reptiles, amphibians, fish, as well as snails, crustaceans, diptera and leeches in the past [82-85], but to our knowledge no acute illness of such hosts upon listerial infection has been reported. Interestingly, the above mentioned poikilothermal animals acidify their stomach or gut content during digestion [86-91], and the resulting low pH may serve as a signal of host entry and virulence gene induction at environmental temperatures.

## Conclusions

Environmental conditions have been identified here as a clue for *L. monocytogenes* virulence. A persistent low pH appears to signal that *L. monocytogenes* has entered a low pH habitat outside animals, resulting in the down regulation of virulence genes and the development of a full ATR. In contrast, a transient low pH at environmental temperature is a signal for this pathogen to potentially have entered a host as evidenced by the increase in cell culture invasion capabilities and faster killing of nematodes. This phenotype is paralleled by an unexpected induction of *prfA* transcription, which is transient and increases with temperature decrease.

## Methods

### Culture conditions and acid shock

If not mentioned otherwise, *L. monocytogenes* EGDe (serovar 1/2a) was used for the experiments. The following *L. monocytogenes* strains were taken from the Weihenstephan *Listeria*Collection (WSLC, ZIEL, Abteilung Mikrobiologie, Technische Universität München, Freising, Germany): WSLC1211 (serovar 3a), WSLC1364 (4b), WSLC1416 (1/2a), WSLC1499 (1/2a), WSLC1685 (4b), WSLC11149 (3a). Cells were grown with shaking (180 rpm) in near neutral BHI (pH 7.2) media at 10°C, 18°C, 25°C or 37°C until an optical density at 600 nm (OD_600nm_) of 0.5 and then exposed to sub lethal acid stress (pH 5.0, BHI adjusted with 5 M HCl). Samples were taken immediately before acid shock as a control (0 min), or 15, 30, 60, 90 and 120 min after acid shock. In each experiment, the control samples were used as reference. Two hours after exposure to pH 5.0, cells were diluted 1:100 into fresh BHI broth at pH 5.2. A sample of cells adapted in this way was taken from this culture when it had reached the middle logarithmic growth phase (OD_600_ = 0.5). A pH of 5.2 was chosen to allow significant growth compared to pH 5.

### RNA isolation

Ten ml cell suspension was added to 20 ml of bacterial RNA Protect (QIAGEN, Hilden, Germany) and sedimented by centrifugation at 5,000 × g for 5 min. The pellets were immediately frozen in liquid nitrogen. Total RNA was isolated using an RNeasy tissue kit (QIAGEN) according to the manufacturer’s recommendations with the following modifications. The frozen cells were resuspended in 700 μl RTL buffer containing 1% β-mercaptoethanol and disrupted thrice in a Ribolyzer (Hybaid, Heidelberg, Germany) using Lysing Matrix B beads (Qbiogene, Heidelberg, Germany) for 45 s at a speed of 6.5 m/s. The cells were cooled on ice between runs. Finally, the tubes were centrifuged for 2 min at 16,100 × g, and the supernatant was removed and processed as described by the manufacturer. DNase digestion was performed using Ambion’s DNA-free (Ambion, Cambridgeshire, United Kingdom) according to the manufacturer’s instructions.

### Real time quantitative qRT-PCR

One μg of isolated total RNA was transcribed with random primers to cDNA by Superscript™ III (Invitrogen, Karlsruhe, Germany). The cDNA was 10-fold serially diluted and real time PCR was performed in an iCycler (BioRad, München, Germany) using ABsolute™ QPCR SYBR^®^ Green Fluorescein mix (ABgene, Hamburg, Germany). Primer for *prfA*-analysis are lmo0200F3 (5′-GTATCACAAAGCTCACGAGT-3′) and lmo0200R3 (5′- TGTATCAATAAAGCCAGACAT-3′). Primer binding to different genetic regions for microarray confirmation are listed in Additional file 3. 16S rRNA was used to normalize the qRT-PCR data as described previously [92].

### Epithelial cell invasion assay

Human colon epithelial cells (Caco-2 cells, ATCC HTB-37), 2.5 × 10^5^ per well, were seeded in a 24-well culture plate and cultivated until infection at 37°C and 5% CO_2_ in RPMI 1640 supplemented with 10% fetal calf serum. Caco-2 cells were not differentiated, and passages 7–13 were used. *L. monocytogenes* was grown in BHI (pH 7.2) at 25°C until OD_600_ = 0.5 and acid-shocked to pH 5.0 using HCl for 30 and 60 min as described above. A sample of bacteria adapted to low pH was obtained as described above. Epithelial cells were washed thrice with PBS/Mg^2+^Ca^2+^ and finally covered for 1 h at 37°C with 500 μl RPMI 1640 containing bacterial cells to a multiplicity of infection of 10. Subsequently, the Caco-2 cells were washed thrice with PBS/Mg^2+^Ca^2+^, incubated for 60 min in 500 μl RPMI 1640 containing 10 μg ml^-1^ gentamycin, and washed with PBS/Mg^2+^Ca^2+^. The infected Caco-2 cells were lysed in 1 ml cold Triton X-100 (0.1%), and intracellular bacteria were quantified by plating appropriate dilutions.

### *C. elegans* infection assays

The infection of *C. elegans* with EGDe was essentially performed as described recently [93]. Briefly, *L. monocytogenes* was grown in BHI to OD_600nm_ = 0.5 at 30°C. An aliquot of this culture was removed as a control, and the remaining cell suspension was acidified to pH 5.0 for 1 h with HCl as described. Ten ml of the acid-shocked and the control culture were centrifuged. The sedimented cells were washed three times in fresh BHI, and the final pellet was spread on nematode growth medium (NGM) agar plates of 8.5 cm diameter. Plates were equilibrated to room temperature (22°C) before use. Twenty *C. elegans* L4 larvae were then transferred onto the listerial lawn. The worms were transferred daily to freshly prepared *L. monocytogenes* as described above, and their survival was monitored for 14 d. Worms were considered dead if they did not respond to touching. The time for 50% of the worms to die (TD_50_) was calculated using the dose–response curve (drc) of the software package R and the function LL.2. The package drc calculates the time at which the inflection point locates at 50% deaths and gives a standard error within a 95% confidence interval estimated by using all given data points [94].

### Microarray analysis

Forty μg of total RNA were reversely transcribed using 9 μg random hexamer primer (Invitrogen), 200U Superscript III RNase H^-^ Reverse Transcriptase (Gibco, Life Technologies), and 2 μl of either Cy3- or Cy5-conjugated dCTP (Amersham Biosciences, Freiburg, Germany). The labeled cDNA was applied to microarray analysis with 70mer oligos (Operon Biotechnologies, Cologne, Germany) spotted on epoxy-slides [39]. Competitive microarray hybridization was performed over night at 50°C in duplicate from two independent experiments, with dye swap arrangements for each experiment. The spots were identified and measured using the software ImaGene (Biodiscovery, El Segundo, CA, USA). After median local background correction, the data were normalized globally using the lowess method [95] implemented in the program. Technical replicates on the same array were combined. Only genes exhibiting substantial changes of RNA levels upon acid treatment (at least threefold signal intensities above local background, expression ratios ≥2 for at least at one time point, more than three valid ratio values) were considered, and the final data were analyzed using GeneSight (Biodiscovery).

## Abbreviations

PTS: Phosphotransferase system; TCS: Two-component system; ATR: Acid tolerance response.

## Competing interests

The authors declare that they have no competing financial interests.

## Authors’ contributions

SS supervised the study, and KN and TMF drafted the manuscript. KN performed RT-PCR and supervised the worm assays, PS conducted the microarray analysis, KS was responsible for cell culture assays. KN and TMF analyzed the data. All authors read and approved the final manuscript.

## Supplementary Material

Additional file 1 **Transcriptomic dynamic of *L. monocytogenes *upon acid shock at two different temperatures.** Click here for file

Additional file 2 **List of genes from *L. monocytogenes *whose knockout led in all cases to attenuation in mouse infection experiments.** Click here for file

Additional file 3 **List of the genes used for the qRT-PCR validation of the *L. monocytogenes *microarray experiments.** Click here for file

Additional file 4 List of genes significantly (p ≤ 0.05) down regulated after acid shock at both 25°C and 37°C. Click here for file
