# Supplementary material for: Acid shock of Listeria monocytogenes at low environmental temperatures induces prfA, epithelial cell invasion, and lethality towards Caenorhabditis elegans
Source: BMC Genomics. 2013 Apr 27;14:285. doi: 10.1186/1471-2164-14-285 (PMC3648428; doi:10.1186/1471-2164-14-285)
Supplement: Additional file 2 — List of genes from L. monocytogenes whose knockout led in all cases to attenuation in mouse infection experiments. [file 1471-2164-14-285-S2.doc]

**Additional file 2** - List of genes from *L. monocytogenes* whose knockout led in all cases to attenuation in mouse infection experiments*

| **Locus tag** | **Gene name** | **Gene description** | **Reference(s)** |
| --- | --- | --- | --- |
| lmo0055 | *purA** | adenylosuccinate synthetase | [1] |
| lmo0137 |  | ABC transporter | [2] |
| lmo0153 | *zinA* | zinc ABC transporter, Zn-binding | [3] |
| lmo0200 | *prfA* | listeriolysin positive regulatory protein | [4] |
| lmo0263 | *inlH* | internalin H | [5-7] |
| lmo0264 | *inlE* | internalin E |
| lmo0433 | *inlA* | internalin A | [8] |
| lmo0434 | *inlB* | internalin B |
| lmo0515 | *-* | universal stress protein | [9] |
| lmo0540 | *-* | penicillin-binding protein | [10] |
| lmo0558 | *pgl* | 6-phosphogluconolactonase | [11] |
| lmo0641 | *frvA* | Fur regulated virulence factor A | [12] |
| lmo0690 | *flaA* | flagellin | [13] |
| lmo0754 | *btlB* | bile acid 7-α-dehydratase | [14] |
| lmo0848 | *-* | putative glutamine transporter | [15] |
| lmo0886 | *dal* | alanine racemase | [16] |
| lmo0931 | *lplA1* | lipoate protein ligase A | [17] |
| lmo0997 | *clpE* | ATP-dependent protease | [18] |
| lmo1273 | *racE*/*rnhB* | RNase H homologue | [15] |
| lmo1295 | *hfq* | RNA-binding protein | [19] |
| lmo1371 | *lpd* | dihydrolipoamide dehydrogenase | [20] |
| lmo1377 | *lisR* | two-component response regulator | [21] |
| lmo1421 | *bilEA* | bile exclusion system | [22] |
| lmo1445 | *zurR* | zinc uptake regulator | [23] |
| lmo1446 | *zurM* | zinc ABC transporter, permease | [3] |
| lmo1580 | *-* | universal stress protein | [9] |
| lmo1634 | *lap* | alcohol dehydrogenase homolog | [24] |
| lmo1666 | *lapB* | peptidoglycan linked protein (LPxTG) | [25] |
| lmo1683 | *perR* | transcription regulator (Fur family), PerR in *B. subtilis* | [26] |
| lmo1695 | *mprF* | multiple peptide resistance factor | [27] |
| lmo1773 | *purB** | adenylosuccinate lyase | [1] |
| lmo1829 | *fbpA* | fibronectin binding protein | [28] |
| lmo2067 | *bsh* | conjugated bile acid hydrolase | [14] |
| lmo2157 | *sepA* | hypothetical protein | [29] |
| lmo2229 | *-* | penicillin-binding protein | [10] |
| lmo2459 | *gap* | glyceraldehyde-3-phosphate dehydrogenase | [30] |
| lmo2468 | *clpP* | ATP-dependent Clp protease proteolytic subunit | [31] |
| lmo2515 | *degU* | response regulator | [32] |
| lmo2549 | *gtcA* | wall teichoic acid glycosylation protein | [33] |
| lmo2558 | *ami* | autolysin, amidase | [34] |
| lmo2589 | *brtA* | bile sensor | [35, 36] |
| lmo2673 | *-* | universal stress protein | [9] |
| lmo2754 | *-* | penicillin-binding protein | [10] |

**sigB* has been tested in a guinea pig model, *purA* and *purB* were tested using a serotype-4 strain in mice.

**References for Additional files**

1. Faith NG, Kim JW, Azizoglu R, Kathariou S, Czuprynski C: **Purine Biosynthesis Mutants (*purA* and *purB*) of Serotype 4b *Listeria monocytogenes* Are Severely Attenuated for Systemic Infection in Intragastrically Inoculated A/J Mice.** *Foodborne Pathog Dis* 2012, **9:**480-486.

2. Schauer K, Geginat G, Liang C, Goebel W, Dandekar T, Fuchs TM: **Deciphering the intracellular metabolism of *Listeria monocytogenes* by mutant screening and modelling.** *BMC Genomics* 2010, **11:**573.

3. Corbett D, Wang J, Schuler S, Lopez-Castejon G, Glenn S, Brough D, Andrew PW, Cavet JS, Roberts IS: **Two zinc uptake systems contribute to the full virulence of *Listeria monocytogenes* during growth *in vitro* and *in vivo*.** *Infect Immun* 2012, **80:**14-21.

4. Chakraborty T, Leimeister-Wächter M, Domann E, Hartl M, Goebel W, Nichterlein T, Notermans S: **Coordinate regulation of virulence genes in *Listeria monocytogenes* requires the product of the *prfA* gene.** *J Bacteriol* 1992, **174:**568-574.

5. Raffelsbauer D, Bubert A, Engelbrecht F, Scheinpflug J, Simm A, Hess J, Kaufmann SH, Goebel W: **The gene cluster *inlC2DE* of *Listeria monocytogenes* contains additional new internalin genes and is important for virulence in mice.** *Mol Gen Genet* 1998, **260:**144-158.

6. Bergmann B, Raffelsbauer D, Kuhn M, Goetz M, Hom S, Goebel W: **InlA- but not InlB-mediated internalization of *Listeria monocytogenes* by non-phagocytic mammalian cells needs the support of other internalins.** *Mol Microbiol* 2002, **43:**557-570.

7. Tsai YH, Orsi RH, Nightingale KK, Wiedmann M: ***Listeria monocytogenes* internalins are highly diverse and evolved by recombination and positive selection.** *Infect Genet Evol* 2006, **6:**378-389.

8. Lingnau A, Domann E, Hudel M, Bock M, Nichterlein T, Wehland J, Chakraborty T: **Expression of the *Listeria monocytogenes* EGD *inlA* and *inlB* genes, whose products mediate bacterial entry into tissue culture cell lines, by PrfA-dependent and -independent mechanisms.** *Infect Immun* 1995, **63:**3896-3903.

9. Seifart Gomes C, Izar B, Pazan F, Mohamed W, Mraheil MA, Mukherjee K, Billion A, Aharonowitz Y, Chakraborty T, Hain T: **Universal stress proteins are important for oxidative and acid stress resistance and growth of *Listeria monocytogenes* EGD-e in vitro and in vivo.** *PLoS One* 2011, **6:**e24965.

10. Guinane CM, Cotter PD, Ross RP, Hill C: **Contribution of penicillin-binding protein homologs to antibiotic resistance, cell morphology, and virulence of *Listeria monocytogenes* EGDe.** *Antimicrob Agents Chemother* 2006, **50:**2824-2828.

11. Crimmins GT, Schelle MW, Herskovits AA, Ni PP, Kline BC, Meyer-Morse N, Iavarone AT, Portnoy DA: ***Listeria monocytogenes* 6-Phosphogluconolactonase mutants induce increased activation of a host cytosolic surveillance pathway.** *Infect Immun* 2009, **77:**3014-3022.

12. McLaughlin HP, Xiao Q, Rea RB, Pi H, Casey PG, Darby T, Charbit A, Sleator RD, Joyce SA, Cowart RE, et al: **A putative P-type ATPase required for virulence and resistance to haem toxicity in *Listeria monocytogenes*.** *PLoS One* 2012, **7:**e30928.

13. O'Neil HS, Marquis H: ***Listeria monocytogenes* flagella are used for motility, not as adhesins, to increase host cell invasion.** *Infect Immun* 2006, **74:**6675-6681.

14. Begley M, Sleator RD, Gahan CG, Hill C: **Contribution of three bile-associated loci, *bsh*, *pva*, and *btlB*, to gastrointestinal persistence and bile tolerance of *Listeria monocytogenes*.** *Infect Immun* 2005, **73:**894-904.

15. Bigot A, Raynaud C, Dubail I, Dupuis M, Hossain H, Hain T, Chakraborty T, Charbit A: **lmo1273, a novel gene involved in *Listeria monocytogenes* virulence.** *Microbiology* 2009, **155:**891-902.

16. Thompson RJ, Bouwer HG, Portnoy DA, Frankel FR: **Pathogenicity and immunogenicity of a *Listeria monocytogenes* strain that requires D-alanine for growth.** *Infect Immun* 1998, **66:**3552-3561.

17. O'Riordan M, Moors MA, Portnoy DA: ***Listeria* intracellular growth and virulence require host-derived lipoic acid.** *Science* 2003, **302:**462-464.

18. Nair S, Frehel C, Nguyen L, Escuyer V, Berche P: **a, a novel member of the HSP100 family, is involved in cell division and virulence of *Listeria monocytogenes*.** *Mol Microbiol* 1999, **31:**185-196.

19. Christiansen JK, Larsen MH, Ingmer H, Søgaard-Andersen L, Kallipolitis BH: **The RNA-binding protein Hfq of *Listeria monocytogenes*: role in stress tolerance and virulence.** *J Bacteriol* 2004, **186:**3355-3362.

20. Sun Y, O'Riordan MX: **Branched-chain fatty acids promote *Listeria monocytogenes* intracellular infection and virulence.** *Infect Immun* 2010, **78:**4667-4673.

21. Cotter PD, Emerson N, Gahan CG, Hill C: **Identification and disruption of *lisRK*, a genetic locus encoding a two-component signal transduction system involved in stress tolerance and virulence in *Listeria monocytogenes*.** *J Bacteriol* 1999, **181:**6840-6843.

22. Sleator RD, Wemekamp-Kamphuis HH, Gahan CG, Abee T, Hill C: **A PrfA-regulated bile exclusion system (BilE) is a novel virulence factor in *Listeria monocytogenes*.** *Mol Microbiol* 2005, **55:**1183-1195.

23. Dowd GC, Casey PG, Begley M, Hill C, Gahan CG: **Investigation of the role of ZurR in the physiology and pathogenesis of *Listeria monocytogenes*.** *FEMS Microbiol Lett* 2012, **327:**118-125.

24. Burkholder KM, Kim KP, Mishra KK, Medina S, Hahm BK, Kim H, Bhunia AK: **Expression of LAP, a SecA2-dependent secretory protein, is induced under anaerobic environment.** *Microbes Infect* 2009, **11:**859-867.

25. Reis O, Sousa S, Camejo A, Villiers V, Gouin E, Cossart P, Cabanes D: **LapB, a novel *Listeria monocytogenes* LPXTG surface adhesin, required for entry into eukaryotic cells and virulence.** *J Infect Dis* 2010, **202:**551-562.

26. Rea RB, Gahan CG, Hill C: **Disruption of putative regulatory loci in *Listeria monocytogenes* demonstrates a significant role for Fur and PerR in virulence.** *Infect Immun* 2004, **72:**717-727.

27. Thedieck K, Hain T, Mohamed W, Tindall BJ, Nimtz M, Chakraborty T, Wehland J, Jansch L: **The MprF protein is required for lysinylation of phospholipids in listerial membranes and confers resistance to cationic antimicrobial peptides (CAMPs) on *Listeria monocytogenes*.** *Mol Microbiol* 2006, **62:**1325-1339.

28. Dramsi S, Bourdichon F, Cabanes D, Lecuit M, Fsihi H, Cossart P: **FbpA, a novel multifunctional *Listeria monocytogenes* virulence factor.** *Mol Microbiol* 2004, **53:**639-649.

29. Weiskirch LM, Paterson Y: ***Listeria monocytogenes*: a potent vaccine vector for neoplastic and infectious disease.** *Immunol Rev* 1997, **158:**159-169.

30. Camejo A, Buchrieser C, Couve E, Carvalho F, Reis O, Ferreira P, Sousa S, Cossart P, Cabanes D: **In vivo transcriptional profiling of *Listeria monocytogenes* and mutagenesis identify new virulence factors involved in infection.** *PLoS Pathog* 2009, **5:**e1000449.

31. Gaillot O, Pellegrini E, Bregenholt S, Nair S, Berche P: **The ClpP serine protease is essential for the intracellular parasitism and virulence of *Listeria monocytogenes*.** *Mol Microbiol* 2000, **35:**1286-1294.

32. Knudsen GM, Olsen JE, Dons L: **Characterization of DegU, a response regulator in *Listeria monocytogenes*, involved in regulation of motility and contributes to virulence.** *FEMS Microbiol Lett* 2004, **240:**171-179.

33. Faith N, Kathariou S, Cheng Y, Promadej N, Neudeck BL, Zhang Q, Luchansky J, Czuprynski C: **The role of *L. monocytogenes* serotype 4b gtcA in gastrointestinal listeriosis in A/J mice.** *Foodborne Pathog Dis* 2009, **6:**39-48.

34. Milohanic E, Jonquieres R, Cossart P, Berche P, Gaillard JL: **The autolysin Ami contributes to the adhesion of *Listeria monocytogenes* to eukaryotic cells via its cell wall anchor.** *Mol Microbiol* 2001, **39:**1212-1224.

35. Quillin SJ, Schwartz KT, Leber JH: **The novel *Listeria monocytogenes* bile sensor BrtA controls expression of the cholic acid efflux pump MdrT.** *Mol Microbiol* 2011, **81:**129-142.

36. Crimmins GT, Herskovits AA, Rehder K, Sivick KE, Lauer P, Dubensky TW, Jr., Portnoy DA: ***Listeria monocytogenes* multidrug resistance transporters activate a cytosolic surveillance pathway of innate immunity.** *Proc Natl Acad Sci U S A* 2008, **105:**10191-10196.

37. Chatterjee SS, Hossain H, Otten S, Kuenne C, Kuchmina K, Machata S, Domann E, Chakraborty T, Hain T: **Intracellular gene expression profile of *Listeria monocytogenes*.** *Infect Immun* 2006, **74:**1323-1338.

38. Joseph B, Przybilla K, Stühler C, Schauer K, Slaghuis J, Fuchs TM, Goebel W: **Identification of *Listeria monocytogenes* genes contributing to intracellular replication by expression profiling and mutant screening.** *J Bacteriol* 2006, **188:**556-568.

39. Milohanic E, Glaser P, Coppée JY, Frangeul L, Vega Y, Vázquez-Boland JA, Kunst F, Cossart P, Buchrieser C: **Transcriptome analysis of *Listeria monocytogenes* identifies three groups of genes differently regulated by PrfA.** *Mol Microbiol* 2003, **47:**1613-1625.

40. Williams T, Bauer S, Beier D, Kuhn M: **Construction and characterization of *Listeria monocytogenes* mutants with in-frame deletions in the response regulator genes identified in the genome sequence.** *Infect Immun* 2005, **73:**3152-3159.

41. Shen A, Higgins DE: **The MogR transcriptional repressor regulates nonhierarchal expression of flagellar motility genes and virulence in *Listeria monocytogenes*.** *PLoS Pathog* 2006, **2:**e30.

42. Williams T, Joseph B, Beier D, Goebel W, Kuhn M: **Response regulator DegU of *Listeria monocytogenes* regulates the expression of flagella-specific genes.** *FEMS Microbiol Lett* 2005, **252:**287-298.

43. Rea R, Hill C, Gahan CG: ***Listeria monocytogenes* PerR mutants display a small-colony phenotype, increased sensitivity to hydrogen peroxide, and significantly reduced murine virulence.** *Appl Environ Microbiol* 2005, **71:**8314-8322.

44. Marr AK, Joseph B, Mertins S, Ecke R, Müller-Altrock S, Goebel W: **Overexpression of PrfA leads to growth inhibition of *Listeria monocytogenes* in glucose-containing culture media by interfering with glucose uptake.** *J Bacteriol* 2006, **188:**3887-3901.

45. Dussurget O, Dumas E, Archambaud C, Chafsey I, Chambon C, Hébraud M, Cossart P: ***Listeria monocytogenes* ferritin protects against multiple stresses and is required for virulence.** *FEMS Microbiol Lett* 2005, **250:**253-261.

46. Faith NG, Kathariou S, Neudeck BL, Luchansky JB, Czuprynski CJ: **A P60 mutant of *Listeria monocytogenes* is impaired in its ability to cause infection in intragastrically inoculated mice.** *Microb Pathog* 2007, **42:**237-241.

47. Schär J, Stoll R, Schauer K, Loeffler DI, Eylert E, Joseph B, Eisenreich W, Fuchs TM, Goebel W: **Pyruvate carboxylase plays a crucial role in carbon metabolism of extra- and intracellularly replicating *Listeria monocytogenes*.** *J Bacteriol* 2010, **192:**1774-1784.

48. Borezee E, Pellegrini E, Berche P: **OppA of *Listeria monocytogenes*, an oligopeptide-binding protein required for bacterial growth at low temperature and involved in intracellular survival.** *Infect Immun* 2000, **68:**7069-7077.

49. Machata S, Tchatalbachev S, Mohamed W, Jansch L, Hain T, Chakraborty T: **Lipoproteins of *Listeria monocytogenes* are critical for virulence and TLR2-mediated immune activation.** *J Immunol* 2008, **181:**2028-2035.
