# Supplementary material for: Acid shock of Listeria monocytogenes at low environmental temperatures induces prfA, epithelial cell invasion, and lethality towards Caenorhabditis elegans
Source: BMC Genomics. 2013 Apr 27;14:285. doi: 10.1186/1471-2164-14-285 (PMC3648428; doi:10.1186/1471-2164-14-285)
Supplement: Additional file 3 — List of the genes used for the qRT-PCR validation of the L. monocytogenes microarray experiments. [file 1471-2164-14-285-S3.doc]

**Additional file 3** - List of the genes used for the qRT-PCR validation of the *L. monocytogenes* DNA-microarray experiments

| **Gene** | **Description** | **Oligonucleo-tide name** | **Sequence** |
| --- | --- | --- | --- |
| lmo0109 | similar to transcriptional regulator AraC family | lmo0109_F  lmo0109_R | TTAATCCTGGCGTGAGTC  GGGAACGTATTTCGAGTG |
| lmo0200 | PrfA | lmo0200F3  lmo0200R3 | GTATCACAAAGCTCACGAGT  TGTATCAATAAAGCCAGACAT |
| lmo0202 | listeriolysin O (*hly*) | lmo0202_F  lmo0202_R | CAAACTGAAGCAAAGGAT  CTTAGGACTTGGAGGCGGAG |
| lmo0679 | FlhB flagellar biosynthetic protein | lmo0679_F  lmo0679_R | TTGATGCCGATTATGGTG  TCAGCCGTTTGAATTGTG |
| lmo0692 | CheA two component sensor histidine kinase | lmo0692_F  lmo0692_R | ATGGTGCCAGTGGACAGT  ATCTGCGCCTTCAATCAC |
| lmo0847 | glutamine ABC transporter | lmo0847_F  lmo0847_R | CTTGAACGCTGGTGCCTAT  TTGCTGCTTCCATTTGTCCT |
| lmo0889 | RsbR positive regulator of SigB activity | lmo0889_F  lmo0889_R | AAAGCAGACTTACTGAATGA  TGCTAGTTTCTTCGTACATT |
| lmo1389 | sugar ABC transporter, ATP binding protein | lmo1389_F  lmo1389_R | GTTAGGAATCAGGAAGGAAT  AATAGCGTAGATTTACCTGC |
| lmo1740 | AA (glutamine) ABC transporter, permease | lmo1740_F  lmo1740_R | TGCGAGGCACGCCATTAC  ACGGCATCCATTCGGTCA |
| lmo1997 | PTS mannose specific enzyme IIA component | lmo1997_F  lmo1997_R | GAAGCGTTCCTCAGTCGT  CGGCATATTCATAAATCCCTAA |
| lmo2434 | glutamate decarboxylase | lmo2434_F  lmo2434_R | AGAAAGCACGAGTATCCC  ATTTCCCTCATCCATCAA |
| lmo2748 | similar to *B. subtilis* stress protein YdaG | lmo2748_F  lmo2748_F | TGGGTGTATTGACATCCGTA  GCCAGATGGTGTATAAAGGG |
| 16S | 16S-rRNA for base calculation in the qRT-PCR | 16S_LmonF1  16S_LmonR1 | AGACACGGCCCAGACTCCT  GATCCGAAAACCTTCTTCATACA |
