# Supplementary material for: Acid shock of Listeria monocytogenes at low environmental temperatures induces prfA, epithelial cell invasion, and lethality towards Caenorhabditis elegans
Source: BMC Genomics. 2013 Apr 27;14:285. doi: 10.1186/1471-2164-14-285 (PMC3648428; doi:10.1186/1471-2164-14-285)
Supplement: Additional file 4 — List of genes significantly (p ≤ 0.05) down regulated after acid shock at both 25°C and 37°C. [file 1471-2164-14-285-S4.doc]

**Additional file 4** - List of genes significantly (p≤0.05) downregulated after acid shock at both 25°C and 37 °C

| **Locus** | **Gene** | **Functional annotation** | **Comments^a^** | **Tempe-rature** | **Min after acid shock^b^** | | | | **Adapta-tion^b, c^** |
| --- | --- | --- | --- | --- | --- | --- | --- | --- | --- |
|  |  |  |  |  | **15** | **30** | **60** | **120** |  |
| lmo0001 | *dnaA* | chromosomal replication initiation protein DnaA | Down regulated intracellularly [37, 38] | 25 °C | 0.7 | 0.4 | 0.2 | 0.1 | 1***^d^** |
|  |  |  |  | 37 °C | 0.4 | 0.3 | 0.3 | 0.5 | 1* |
| lmo0178 |  | similar to xylose repressor | Negatively regulated by PrfA [39], down regulated intracellularly [38] | 25 °C | 0.8 | 0.4 | 0.2 | 0.2 | 1.6 |
|  |  |  |  | 37 °C | 0.4 | 0.4 | 0.5 | 1* | 1* |
| lmo0394 |  | similar to *L. monocytogenes* extracellular P60 protein, peptidoglycan-hydrolase | Down regulated intracellularly [38] | 25 °C | 0.3 | 0.1 | 0.03 | 0.02 | 1.6 |
|  |  |  |  | 37 °C | 1* | 0.1 | 0.02 | 0.02 | 1* |
|  |  |  |  | 37 °C | 0.7 | 1* | 0.4 | 1* | 2.4 |
| lmo0644 |  | similar to conserved hypothetical proteins | Repressed by DegU, DegU is necessary for full virulence [32, 40] | 25 °C | 0.9 | 0.8 | 0.4 | 0.3 | 1* |
|  |  |  |  | 37 °C | 0.4 | 0.5 | 0.6 | 1* | 1.4 |
| lmo0675 |  | flagellar switch protein | Down regulated in infection due to MogR temperature repression [41], repressed by DegU [42] | 25 °C | 0.5 | 0.2 | 0.1 | 0.1 | 1* |
|  |  |  |  | 37 °C | 0.4 | 0.5 | 0.6 | 0.7 | 1* |
| lmo0888 |  | similar to *B. subtilis* YdcE protein | Down regulated intracellularly [37] | 25 °C | 0.4 | 0.3 | 0.4 | 0.6 | 1* |
|  |  |  |  | 37 °C | 0.2 | 0.3 | 0.3 | 0.2 | 1* |
| lmo0970 |  | enoyl-acyl-carrier protein reductase | Down regulated intracellularly [37] | 25 °C | 1* | 0.6 | 0.2 | 0.1 | 1* |
|  |  |  |  | 37 °C | 1* | 0.2 | 0.4 | 0.4 | 1.5 |
| lmo1081 |  | glucose-1-phosphate thymidyl transferase | Down regulated intracellularly [37] | 25 °C | 1* | 0.8 | 0.3 | 0.1 | 1* |
|  |  |  |  | 37 °C | 0.4 | 0.3 | 0.4 | 0.6 | 1* |
| lmo1084 |  | dTDP-4-dehydrorhamnose reductase | Down regulated intracellularly [37] | 25 °C | 1* | 1* | 0.4 | 0.2 | 1* |
|  |  |  |  | 37 °C | 0.6 | 0.5 | 1* | 0.5 | 1* |
| lmo1254 |  | similar to α,α-phosphotrehalase | Down regulated in the vacuolar compartment and upregulated in the cytosolic compartment of the host cell [37] | 25 °C | 1* | 0.9 | 0.4 | 0.2 | 0.4 |
|  |  |  |  | 37 °C | 0.4 | 0.3 | 0.1 | 0.1 | 1* |
| lmo1544 | *minD* | highly similar to cell division inhibitor (septum placement) | Down regulated intracellularly [37, 38] | 25 °C | 0.3 | 0.2 | 0.1 | 0.2 | 1.5 |
|  |  |  |  | 37 °C | 0.5 | 0.5 | 0.5 | 1* | 1* |
| lmo1545 | *minC* | similar to cell-division inhibition (septum placement) | Down regulated intracellularly [37, 38] | 25 °C | 0.3 | 0.1 | 0.1 | 0.2 | n.a.**^e^** |
|  |  |  |  | 37 °C | n.a.**^e^** | 0.3 | 0.4 | n.a.**^e^** | 1.5 |
| lmo1604 |  | similar to 2-cys peroxiredoxin | Down regulated by PerR and intracellularly [38, 43] | 25 °C | 0.9 | 0.9 | 0.8 | 0.5 | 0.7 |
|  |  |  |  | 37 °C | 1* | 0.4 | 0.2 | 0.3 | 0.4 |
| lmo1808 | *fabD* | malonyl CoA-acyl carrier protein transacylase | Down regulated intracellularly [37] | 25 °C | 1* | 0.7 | 0.2 | 0.1 | 1* |
|  |  |  |  | 37 °C | 1* | 0.2 | 0.3 | 0.4 | 1* |
| lmo1809 | *plsX* | involved in fatty acid/phospholipid synthesis | Down regulated by PrfA [44] | 25 °C | 1* | 0.7 | 0.3 | 0.4 | 1* |
|  |  |  |  | 37 °C | 0.4 | 0.4 | 0.3 | 1* | 1* |
| lmo1995 | *dra* | glucosamine-fructose-6-phosphate aminotransferase, opine catabolism | Down regulated by Fri [45] | 25 °C | 1* | 0.7 | 0.3 | 0.6 | 0.7 |
|  |  |  |  | 37 °C | 0.5 | 0.3 | 0.4 | 0.4 | 1* |
| lmo2020 | *divIVA* | cell-division initiation protein (septum placement) | Down regulated intracellularly [37] | 25 °C | 0.7 | 0.5 | 0.3 | 0.4 | 1* |
|  |  |  |  | 37 °C | 1* | 0.3 | 0.4 | 0.4 | 1* |
| lmo2032 | *ftsZ* | cell-division initiation protein FtsZ | Down regulated intracellularly [37, 38] | 25 °C | 1* | 0.6 | 0.3 | 0.4 | 1* |
|  |  |  |  | 37 °C | 0.3 | 0.3 | 0.4 | 0.5 | 1* |
| lmo2039 | *pbpB* | similar to penicillin-binding protein | Down regulated intracellularly [38] | 25 °C | 0.6 | 0.3 | 0.2 | 0.2 | 1* |
|  |  |  |  | 37 °C | 0.2 | 0.3 | 0.4 | 1* | 1.3 |
| lmo2202 |  | 3-oxoacyl-[acyl-carrier-protein] synthase III | Down regulated intracellularly [37, 38] | 25 °C | 0.6 | 0.3 | 0.1 | 0.2 | 1* |
|  |  |  |  | 37 °C | 0.3 | 0.4 | 1* | 1* | 1.5 |
| lmo2506 | *ftsX* | highly similar to cell-division protein FtsX | Down regulated intracellularly [37, 38] | 25 °C | 0.5 | 0.2 | 0.1 | 0.1 | 1* |
|  |  |  |  | 37 °C | 1* | 0.1 | 0.2 | 0.1 | 1* |
| lmo2507 | *ftsE* | highly similar to cell-division ATP-binding protein FtsE | Down regulated intracellularly [37, 38] | 25 °C | 0.5 | 0.2 | 0.04 | 0.04 | 1.6 |
|  |  |  |  | 37 °C | 1* | 0.1 | 0.1 | 0.1 | 1* |
| lmo2691 | *murA*/ *namA* | autolysin, N-acetylmuramidase | Down regulated intracellularly [37] | 25 °C | 1.4 | 1* | 0.7 | 0.4 | 1* |
|  |  |  |  | 37 °C | 0.6 | 0.5 | 0.3 | 0.3 | 1* |
| lmo2718 | *cydA* | cytochrome D ubiquinol oxidase subunit I | Down regulated intracellularly [37] | 25 °C | 1* | 1.6 | 0.9 | 0.2 | 0.5 |
|  |  |  |  | 37 °C | 0.7 | 0.3 | 0.3 | 0.3 | 0.3 |
|  |  |  |  |  |  |  |  |  |  |
|  |  |  |  |  |  |  |  |  |  |
| lmo0582 | *iap* | P60 extracellular protein, invasion associated protein | Mutant is attenuated in intragastrically inoculated mice [46] | 25 °C | 1.6 | 0.8 | 0.3 | 0.4 | 1* |
|  |  |  |  | 37 °C | 0.5 | 0.4 | 0.4 | 0.4 | 1.4 |
| lmo0847 |  | Putative glutamine transporter | Inactivation impairs virulence [15] | 25 °C | 0.8 | 0.5 | 1* | 0 | 0.2 |
|  |  |  |  | 37 °C | 1* | 0.2 | 1* | 0.2 | 0.1 |
| lmo1072 | *pycA* | pyruvate carboxyl transferase | Mutant attenuated in mice [47] | 25 °C | 1* | 0.5 | 0.2 | 0.2 | 1* |
|  |  |  |  | 37 °C | 0.3 | 0.3 | 0.4 | 0.5 | 1* |
| lmo2196 | *oppA* | oligopeptide-binding protein | Involved in intracellular survival [48] | 25 °C | 1* | 0.9 | 0.9 | 0.3 | 0.8 |
|  |  |  |  | 37 °C | 1* | 0.4 | 0.2 | 0.2 | 0.5 |
| lmo2482 | *lgt* | Prolipoprotein diacylglyceryl transferase | Critical for virulence [49] | 25 °C | 0.8 | 0.5 | 0.2 | 0.1 | 1* |
|  |  |  |  | 37 °C | 1* | 0.3 | 0.3 | 1* | 1.4 |
|  |  |  |  |  |  |  |  |  |  |

**^a^** Comments are based on the given reference(s).

**^b^** In case of up regulations, the integer digits are significant; for downregulated genes, the first post decimal digit is significant.

**^c^** The pH for adaptation was 5.2 instead of 5.0 as for all other time points.

**^d^** 1*: No significant difference (p > 0.05) between experiment and control and therefore set to 1.

**^e^** n.a.: Not available.

**See Additional file 2 for references.**
